# Association of lipid composition and unsaturated fatty acids of VLDL with atrial remodeling in metabolic syndrome

**DOI:** 10.1038/s41598-023-33757-0

**Published:** 2023-04-21

**Authors:** Hsiang-Chun Lee, Wei-Chung Cheng, Wen-Lung Ma, Yu-Hsun Lin, Shyi-Jang Shin, Yi-Hsiung Lin

**Affiliations:** 1 Division of Cardiology, Department of Internal Medicine, Kaohsiung Medical University Hospital, Kaohsiung Medical University, 100 Tzyou 1st Rd, Kaohsiung, 807 Taiwan; 2 Department of Internal Medicine, School of Medicine, College of Medicine, Kaohsiung Medical University, Kaohsiung, Taiwan; 3 Lipid Science and Aging Research Center, College of Medicine, Kaohsiung Medical University, Kaohsiung, Taiwan; 4 Institute/Center of Medical Science and Technology, National Sun Yat-Sen University, Kaohsiung, Taiwan; 5 Graduate Institute of Animal Vaccine Technology, National Pingtung University of Science and Technology, Pingtung, Taiwan; 6 PhD Program for Cancer Molecular Biology and Drug Discovery, China Medical University and Academia Sinica, Taichung, Taiwan; 7 Research Center for Cancer Biology, China Medical University, Taichung, Taiwan; 8 Graduate Institute of Biomedical Sciences, and Graduate Institution of Cancer Biology, School of Medicine, China Medical University, Taichung, Taiwan; 9 Department of Medical Research, China Medical University Hospital, Taichung, Taiwan; 10 Department of Nursing, Asia University, Taichung, Taiwan; 11 Grander Clinic, Kaohsiung, Taiwan

**Keywords:** Cardiovascular biology, Endocrine system and metabolic diseases, Cardiology, Endocrinology, Medical research

## Abstract

Subjects with metabolic syndrome (MetS) commonly have atrial remodeling, which indicates a risk for atrial fibrillation. This study determined MetS-related changes in lipid components in very-low-density lipoprotein (VLDL), which has been shown to cause atrial remodeling, the effect of statins on these changes, and the correlation between atrial remodeling and VLDL lipid compositions. Blood samples were collected from 12 non-MetS and 27 sex- and age-matched MetS subjects. Fourteen patients with MetS (MetS-off statin) discontinued statin therapy 14 days before the study, while the remaining 13 remained on it (MetS-on statin). The VLDLs were isolated and processed for mass-based lipid profiling. Lipidomic analyses were performed and associated with atrial remodeling markers measured using standard echocardiography and electrocardiography. Compared with the VLDL components of the non-MetS group, glucosyl/galactosyl ceramide, lyso-phosphatidylcholine, lyso-phosphatidylethanolamine, and triglycerides were enriched in the MetS-off statin group. Statin therapy attenuated all abnormally abundant lipid classes in MetS, except for triglycerides. In addition, lyso-phosphatidylcholine, lyso-phosphatidylethanolamine, and triglycerides were significantly correlated with atrial dilatation, and the latter two were also correlated with the PR interval. Enrichment of double bonds, which indicate unsaturated fatty acids, was also significantly correlated with atrial remodeling and P-wave duration. This study suggests that the pathological lipid payload of MetS-VLDL may contribute to atrial remodeling in patients.

## Introduction

Metabolic syndrome (MetS) is a cluster of disorders that is strongly linked to increased risks and adverse outcomes of cardiovascular diseases, including atrial fibrillation (AF), the most prevalent arrhythmia^1–5^. The pathogenic mechanisms by which MetS leads to atrial remodeling and AF development have also been clearly identified^4–8^. Central obesity, an important feature of MetS, is strongly correlated with the incidence of AF and is associated with a 50% increase in AF risk^9^. Nevertheless, the contribution of dyslipidemia to AF has been contradictory in clinical observational studies. Possible explanations include different study designs, populations, age ranges, and sex differences in lipid metabolism. Total cholesterol and low-density lipoprotein (LDL) cholesterol in a recent meta-analysis are inversely correlated with incident AF^10^. Nevertheless, the pleiotropic effects of statins, the most commonly used lipid-lowering drugs for preventing and treating AF, have been consistently reported^11, 12^.

Independent of LDL, the pathogenic roles of very low-density lipoproteins (VLDL) in the cardiometabolic disorders, including atrial myopathy and AF have been noticed^13–16^. In previous studies, the in vivo effects of VLDL extracted from patients with MetS were examined in mice and showed excess lipid accumulation associated with apoptosis in the atria with greater left atrial size and vulnerability to AF^17^. Mechanistic findings include delayed intracardiac conduction velocities, modulated gap junctions^18^, disrupted calcium regulation, and derangements in sarcomere proteins^19^. The aforementioned findings were not observed with VLDL isolated from healthy volunteers without MetS, suggesting that the alteration of lipid components in the VLDL of MetS may be responsible for its ability to promote lipotoxicity in the atrium.

AF in MetS usually develops insidiously and occurs until the structural and electrical changes within the atria have progressed over a long time (mostly years to a couple of decades)^20–22^. Structural remodeling of the atrium is resulted from myocardial inflammation, apoptosis, and increased tissue fibrosis^23^ and demonstrated by the hallmark left atrial (LA) dilation in the echocardiographic examination. The increased LA diameter is also a significant marker of AF risk and outcomes^24^. Electrical remodeling of the atrium which is resulted from expressional and functional changes in ionic channels, delayed conduction velocity, and abnormally triggered electrical activity^25^, can be reflected by the P-wave duration and PR interval of electrocardiography^26, 27^. In this study, echocardiography and electrocardiography were used to evaluate the structural and the electrical remodeling of the atrium respectively.

This study performed lipidomic analysis of VLDLs isolated from 12 non-MetS and 27 sex/age-matched MetS subjects. Approximately half of the participants with MetS discontinued statin therapy. The objectives of this study were as follows: first, to determine the changes in lipid species in the VLDL of MetS; second, to determine if statins can improve the lipid component changes in VLDL of MetS; and finally, to determine if there is any correlation between the lipid component of VLDL and atrial remodeling in MetS.

## Methods

### Study subjects

To determine the differences in lipid species of VLDL between MetS and non-MetS subjects and to determine the lipid-lowering drug, that is, statins, on lipid species of VLDL, this study enrolled participants at a single medical center. Those with any of the following were excluded: significant coronary artery disease, myocardial infarction, congenital heart diseases, heart failure, significant heart valve diseases, cerebrovascular diseases, cancers, insulin therapy, and pregnant or breastfeeding women. Those who met any 3 of the following 5 criteria were diagnosed with MetS: (1) central obesity (waist circumference ≥ 80 cm for women and ≥ 90 cm for men); (2) elevated blood pressure (BP) (systolic BP ≥ 130 mmHg or diastolic BP ≥ 85 mmHg or treatment of previously diagnosed hypertension); (3) elevated plasma fasting glucose (≥ 100 mg/dL or diagnosed type 2 diabetes mellitus); (4) elevated plasma fasting triglyceride levels (≥ 150 mg/dL or on triglyceride (TAG)-lowering treatment); and (5) reduced plasma high-density lipoprotein cholesterol (< 50 mg/dL for women and < 40 mg/dL for men). Among the participants, 12 non-MetS and 27 MetS subjects were selected based on age and sex matching. All MetS patients had received regular medicine (including statin) over 1 year. Fourteen patients with MetS (the MetS-off statin group) were requested to discontinue all lipid-lowering drugs, that is, statins, 14 days prior to the study visit for sample and data collection, while the remaining 13 patients with MetS were informed to continue ordinary medicine, including statins (the MetS-on statin group). The study protocol was reviewed and approved by the Kaohsiung Medical University Hospital Institutional Review Board (IRB) (KMUHIRB-E(I)-20170256) and registered with trial registration number ISRCTN 69295295 (retrospectively registered on June 9, 2020). All subjects signed an informed consent form before participating. The study adhered to the principles of the Declaration of Helsinki. Each participant also underwent measurements of height, body weight, blood pressure, heart rate, and abdominal and hip circumferences at the study visit. Medical records, if available, were reviewed, and medication use was recorded.

### VLDL isolation and lipid profiling for lipidome analysis

All study subjects were instructed to fast before prior midnight until 20 mL venous blood draws were completed and collected in BD VACUETTE^®^ EDTA tubes (Becton, Dickinson and Company, Franklin Lakes, NJ, USA) for subsequent VLDL isolation as described previously^14^. Briefly, the blood samples were centrifuged and separated from blood cells, and plasma was maintained for high-speed centrifugation at 10,000 rpm for 1 h to remove the upper chylomicrons. The ultracentrifuge at 40,000 rpm at 4 °C for 24 h resulted in separated VLDL (density between 1.006 and 1.063 g/mL) on the top. The VLDL samples were transported to lipid profiling using Lipotype GmbH (Dresden, Germany)^28^.

### Nomenclature

Lipid names and abbreviations are listed in Abbreviations. Lipid species were annotated as follows: (Lipid class) − (number of carbon atoms in fatty acids): (number of double bonds in fatty acids); (number of hydroxyl groups in long-chain base and fatty acids moiety).

### Lipid extraction for MS lipidomics

Lipids were extracted using one-step procedure with methyl tert-butyl ether/methanol (7:2, v/v) was used as a solvent^16^. The procedures and details of the shotgun lipidomics were followed as those presented by Surma et al.^29^. The m/z values for all measured lipids (an EXCEL file) can be found in the supplementary material. Samples were spiked with a mixture of internal lipid standard mixture containing the following: cardiolipin (CL), 16:1/15:0/15:0/15:0; ceramide (Cer), 18:1;2/17:0; diacylglycerol (DAG), 17:0/17:0; glucosyl/galactosyl ceramide (HexCer), 18:1;2/12:0; lysospecies lyso-phosphatidic acid (LPA), 17:0; lyso-phosphatidylcholine (LPC), 12:0; lyso-phosphatidylethanolamine (LPE), 17:1; lyso-phosphatidylglycerol (LPG), 17:1; lyso-phosphatidylinositol (LPI), 17:1; lyso-phosphatidylserine (LPS), 17:1; PA, 17:0/17:0; PC, 17:0/17:0; PE, 17:0/17:0; PG, 17:0/17:0; PI, 16:0/16:0; PS, 17:0/17:0; Chol ester (CE), 20:0; sphingomyelin (SM), 18:1;2/12:0; TAG, 17:0/17:0/17:0, and Chol. After extraction, the organic phase was transferred to an infusion plate for dried in a speed vacuum concentrator. Each first-step dry extract was resuspended in 7.5 mM ammonium acetate in chloroform/methanol/propanol (1:2:4, v/v/v), and each second-step dry extract was resuspended in 33% ethanol solution of methylamine/chloroform/methanol (0.003:5:1, v/v/v). All liquid handling steps were performed using the Hamilton Robotics STARlet robotic platform with the Anti-Droplet Control feature for organic solvent pipetting.

### MS data acquisition

Samples were analyzed by direct infusion on a Q-Exactive mass spectrometer (Thermo Fisher Scientific Inc., Waltham, MA, USA) equipped with a TriVersa NanoMate ion source (Advion BioSciences, Inc. Ithaca, NY, USA). Samples were analyzed in both the positive and negative ion modes with a resolution of 280,000 at m/z = 200 for MS and was 17,500 for MS/MS experiments in a single acquisition. MS/MS was triggered by an inclusion list encompassing corresponding MS mass ranges scanned in 1-Da increments. Both MS and MS/MS data were combined to monitor CE, DAG, and TAG ions as ammonium adducts; PC and PC O- as acetate adducts; and CL, PA, PE, PE O-, PG, PI, and PS as deprotonated anions. Only MS was used to monitor LPA, LPE, LPE O-, LPI, and LPS as deprotonated anions; Cer, HexCer, SM, LPC, and LPC O- as acetate adducts; and Chol as an ammonium adduct of an acetylated derivative^29^.

### Data analysis and postprocessing

Lipid identification was performed using LipotypeXplorer unprocessed mass spectra. With the MS-only mode, lipid identification was performed according to the molecular masses of intact molecules. With MS/MS mode, the identification of the collision-induced fragmentation of lipid molecules was performed according to both the intact masses and the fragment masses. Identification signals were filtered according to mass accuracy, occupation threshold, noise, and background prior to normalization and statistical analysis^30^. Intensities of lipids with identity were stored in an optimized lipidomic database for the particular structure. The acquired intensity of lipid molecules was translated to lipid amounts by normalization to class-specific internal standards^31^. The total amount of the specific lipid class was the summation of all individual lipid molecules (species or subspecies, in p-moles) of a given lipid class. The relative amounts of the lipid classes were normalized to the total lipid amount in mol.% per total lipids.

### Lipidomic data processing

The processed lipidomic data were analyzed using LipidSig^32^. In brief, the lipid profiling data of each sample were scale-normalized to the total amount of lipids. Lipid classes with larger than twofold changes between groups were identified as significantly changed in the presence of MetS or by the use of lipid-lowering drugs. The enrichment of the changed lipids in each lipid class (such as PC, PE, and LPC) was examined using Fisher’s exact test for significance. In addition, Spearman’s correlation was used to examine the correlations between lipid amounts and clinical factors.

### Laboratory testing for biochemical data

Biochemical data were obtained from the Department of Laboratory Medicine at Kaohsiung Medical University Hospital according to standard laboratory procedures. Technicians were blinded to the participants’ identities and clinical conditions. The laboratory data, including glucose, hemoglobin A1c (HbA1c), total Chol, TAG, VLDL, LDL-Chol, HDL-Chol, alanine aminotransferase (ALT), creatinine, and uric acid, were collected.

### Echocardiographic assessment

Transthoracic echocardiography was performed for the measurement of left atrium (LA) diameter, maximum volume, and minimum volume by an experienced cardiologist using a cardiac probe (Vivid 9E; General Electric Medical Systems, Horten, Norway), according to the standards of the American Society of Echocardiography^33^. LA volumes and total emptying fraction (EF) of the LA were derived using the modified Simpson’s method. Raw data were assessed while the examiners were blinded to clinical and lipid data.

### Measurement of electrocardiographic (ECG) parameters

Twelve-lead ECG was performed by medical technicians at the study visit. One experienced technician who was blinded to clinical information and data performed the measurement of ECG parameters, including P wave durations, PR intervals, QRS width, QTc intervals in lead II, and the duration and terminal force of P waves in lead V1^34^. Only regular sinus rhythm was included for measurement, and any arrhythmias, bundle branch block, obvious ST-T abnormalities, and atrioventricular blocks were discarded.

### Statistical analysis

All continuous variables (mean ± standard deviation) in demographic and clinical data were compared among the three groups: non-MetS, MetS-off statin, and MetS-on statin using ordinary one-way ANOVA followed by multiple comparisons with Tukey’s test. The results were considered statistically significant with a *P* value of < 0.05. Statistical tools included SPSS statistical software (version 22; IBM Corp., Armonk, NY, USA), SAS 9.4 software (SAS Institute Inc., Cary, NC, USA), and GraphPad Prism (version 9; GraphPad Software, Inc., San Diego, CA, USA) software system. Spearman’s correlation was performed to determine the correlation between specific lipids and each parameter of cardiac remodeling (for the atrium and the ventricle) and MetS.

## Results

### Characteristics of study subjects

Among the 12 non-MetS subjects, 14 MetS-off statin subjects and 13 MetS-on statin subjects, sex, and age were matched (Table 1). There was no difference in the presence of hypertension or diabetes mellitus. Compared with the non-MetS group, the two MetS groups had greater obesity parameters, including BMI and waist and hip circumferences. Similarly, systolic and diastolic blood pressures were higher in the MetS groups. There were no significant differences in markers for renal function creatinine or liver function ALT levels. In common lipid profiles, while cholesterol levels were similar among groups, the MetS-off statin group had significantly lower HDL-C (37.0 ± 7.9 mg/dL vs. non-MetS 63.8 ± 15.8 mg/dL, *P* < 0.0001), higher triglycerides (235.1 ± 177.0 mg/dL vs. non-MetS 88.7 ± 21.1 mg/dL, *P* = 0.006) values and higher uric acid (6.4 ± 1.4 mg/dL vs. non-MetS 5.1 ± 0.9 mg/dL, *P* = 0.0234).

**Table 1:** Characteristics of study subjects.

	Non-MetS (n = 12)	MetS-off statin (n = 14)	MetS-on statin (n = 13)	*P* value
Age (years)	48.4 ± 7.8	54.0 ± 8.8	54.0 ± 7.8	0.1528
Men:women (n:n)	7:5	7:7	8:5	
BMI (kg/m^2^)	21.5 ± 2.4	30.1 ± 4.5*	30.2 ± 2.1^¶^	< 0.0001
Waist circumference (cm)	78.9 ± 8.0	99.5 ± 9.4*	103.8 ± 9.2^¶^	< 0.0001
Hip circumference (cm)	95.1 ± 4.0	103.9 ± 9.0*	105.5 ± 6.9^¶^	0.0015
Hypertension, n (%)	0 (0)	11 (84.6)	13 (100)	< 0.0001
Diabetes mellitus, n (%)	0 (0)	8 (61.5)	9 (69.2)	0.0010
Systolic BP (mmHg)	113.3 ± 13.8	141.1 ± 15.6*	150.1 ± 19.0^¶^	< 0.0001
Diastolic BP (mmHg)	69.0 ± 8.5	86.5 ± 10.2*	93.8 ± 11.3^¶^	< 0.0001
Fasting glucose (mg/dL)	87.0 ± 7.1	119.7 ± 26.9*	110.3 ± 25.9^¶^	0.0020
HbA1c (%)	5.5 ± 0.3	6.7 ± 1.5	6.3 ± 0.7	0.0712
Total cholesterol (mg/dL)	169.2 ± 25.2	183.6 ± 34.6	168.6 ± 25.4	0.3291
Triglyceride (mg/dL)	88.7 ± 21.1	235.1 ± 177.0*	173.3 ± 60.5	0.0084
VLDL (mg/dL)	2.7 ± 3.1	27.1 ± 25.1*	18.7 ± 9.3^¶^	0.0018
LDL-Cholesterol (mg/dL)	105.4 ± 25.4	119.5 ± 28.6	105.3 ± 23.1	0.2761
HDL-Cholesterol (mg/dL)	63.8 ± 15.8	37.0 ± 7.9*	44.6 ± 9.4^¶^	< 0.0001
ALT (IU/L)	22.6 ± 10.6	30.8 ± 14.6	24.9 ± 9.8	0.1727
Creatinine (µIU/mL)	0.78 ± 0.11	0.78 ± 0.16	0.76 ± 0.17	0.9644
Uric acid (mg/dL)	5.1 ± 0.9	6.4 ± 1.4*	5.6 ± 1.2	0.0292

*MetS* metabolic syndrome, *DC* discontinued, *BMI* body mass index, *BP* blood pressure, *ALT* alanine aminotransferase, *VLDL* very-low-density lipoprotein, *LDL* low-density lipoprotein, *HDL* high-density lipoprotein.

*P < 0.05, MetS-off statin group versus Non-MetS group;

^¶^*P* < 0.05, MetS-on statin group versus Non-MetS group.

### Structural remodeling of the LA demonstrated by echocardiography

**Figure 1:**
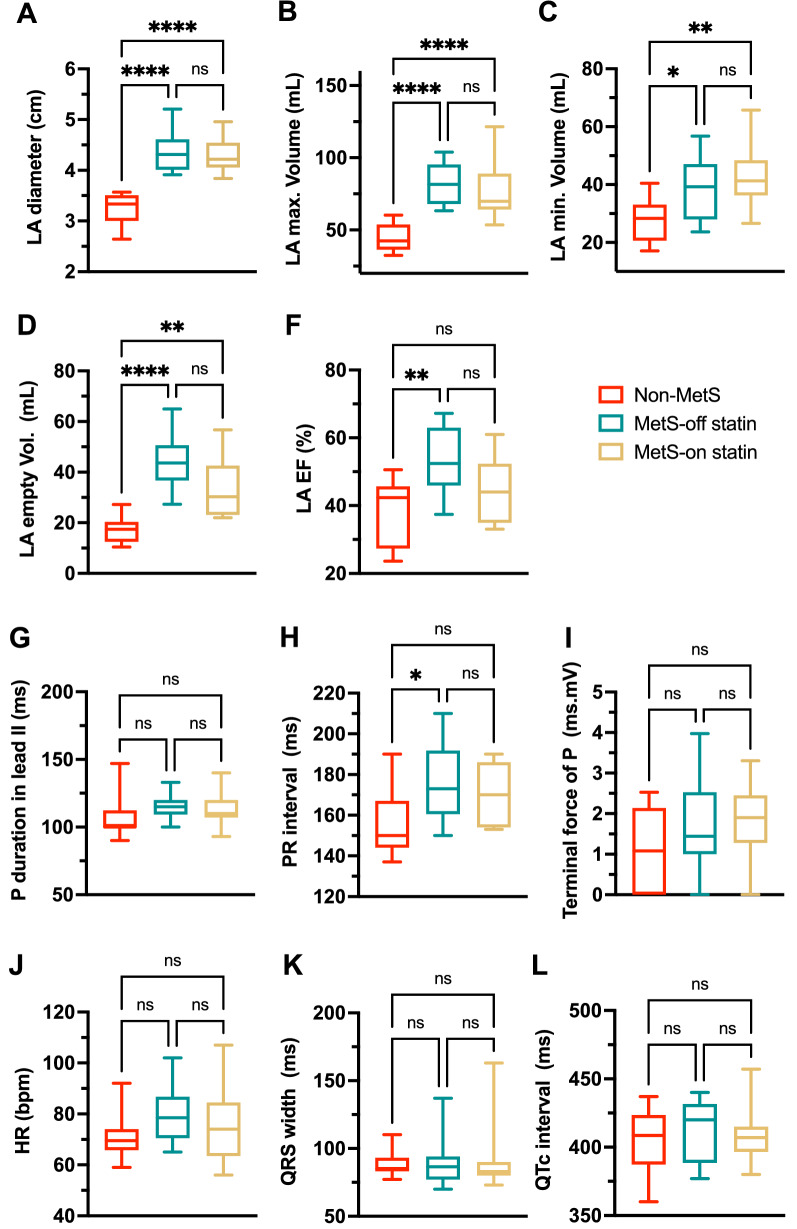
Atrial size, function, and electrical activity measurements in echocardiography and electrocardiography. (**A**–**C**) LA size (in diameter and volumes) was significantly larger in both MetS (-off statin and -on statin) groups than in the non-MetS group. (**D**–**F**) The MetS groups had a larger LA emptying volume. The emptying fraction (EF) was significantly larger in the MetS-off statin group. *P < 0.05, **P < 0.01, and ****P < 0.0001. (**G**–**I**) P wave duration and PR interval, QRS, and QTc interval in lead II, and the terminal force of the P wave in lead V1 are compared among groups. The MetS-off statin group had a significantly prolonged PR interval in lead II. *P < 0.05. (**J**–**L**) Comparison of heart rate, QRS width and QTc interval among groups.

Echocardiography was performed for each study subject to measure LA size in diameter, volumes, and function in the EF, which indicates atrial contractility (Fig. 1A–F). Compared with the non-MetS group, the MetS groups, whether off or on statin, had a significantly larger LA chamber size with greater diameters (LA diameter, non-MetS 3.2 ± 0.3 cm vs. MetS-off statin 4.4 ± 0.4 cm vs. MetS-on statin 4.3 ± 0.3 cm, *P* < 0.0001) and greater volumes (LA maximum volume, non-MetS 45.2 ± 9.5 mL vs. MetS-off statin 81.9 ± 13.9 mL vs. MetS-on statin 77.5 ± 18.9 mL, *P* < 0.0001; LA minimum volume, non-MetS 28.1 ± 7.5 mL vs. MetS-off statin 39.3 ± 10.8 mL vs. MetS-on statin 42.0 ± 11.3 mL, *P* = 0.0065). Interestingly, the EF of LA was significantly greater in the MetS-off statin group than in the non-MetS group (LAEF, non-MetS 38.1 ± 9.5% vs. MetS-off statin 53.3 ± 9.7%, *P* = 0.0041).

### Atrial electrical remodeling revealed by electrocardiography

Electrocardiography was performed on each study subject to measure various P-wave parameters (Fig. 1). The PR interval was significantly wider in the MetS-off statin group than in the non-MetS group (176.1 ± 19.0 ms vs. 156.2 ± 15.4 ms, *P* = 0.0014), whereas the P wave duration (in leads II and V1), terminal force of P waves, QRS width and QTc interval were not significantly different among groups (Fig. 1G–L).

### Lipid components of VLDL are different between non-MetS and MetS groups, and the changes are blunted by statin treatment

**Figure 2:**
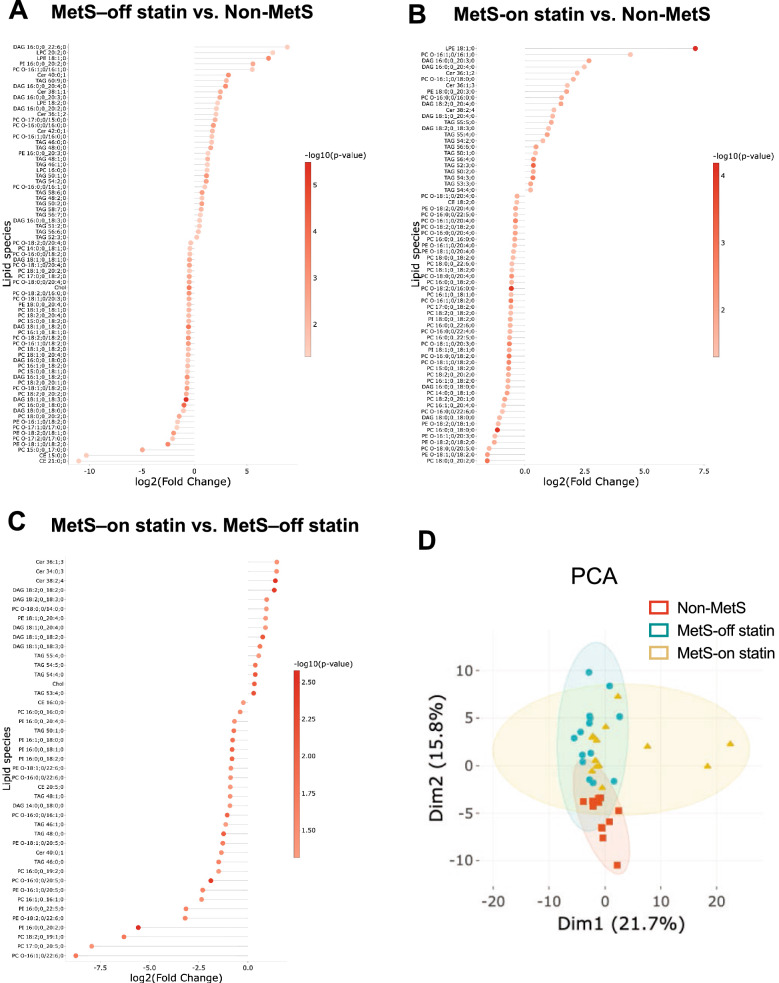
Comparison of lipid species in VLDLs of non-MetS, MetS-off statin, and MetS-on statin groups. Lipid species with at least a twofold change were identified as significantly different between groups, including those belonging to diacylglycerol (DAG), lyso-phosphatidylcholine (LPC), lyso-phosphatidylethanolamine (LPE), (PI), ceramide (Cer), triglyceride (TAG), phosphatidylcholine (PC), cholesterol (Chol), and ether derivatives of PE (PE O-), PC (PC O-), and cholesteryl ester (CE). Principal component analysis (PCA) shows differences among the three groups.

The lipid components of VLDL were determined using mass spectrometry. In the comparison analysis, the most different lipid species were found between non-MetS and MetS-off statins: DAG 16:0, LPC, LPE, PI, Cer, and TAG were significantly higher in the MetS-off statin group than in the non-MetS group, whereas CE, PC, PC-O, PE-O, and DAG 18:0 were significantly lower in the MetS-off statin group than in the non-MetS group (Fig. 2A). Compared to non-MetS, the MetS-on statin had lipid species in VLDL higher with LPE, PC-O, DAG 16:0, Cer, PE, and TAG but lower with PC, PC-O, PE-O, DAG 18:0, and PI (Fig. 2B). The comparison between the two MetS, the -off and -on statin groups, indicated the effect of statins on lipid species of VLDL that PC-O, PC, PE-O, PI, long chain TAG (46:0, 46:1, 48:0, 48:1, and 50:1), and CE were significantly reduced but Cer, DAG 18:0–2, PE, Chol, and TAG 54:4, 54:5, and 55:4 were significantly increased (Fig. 2C). PCA demonstrated the patterns of lipid species in VLDL among the three groups (Fig. 2D).

### Changes in lipid structural and functional categories in VLDL of MetS

**Figure 3:**
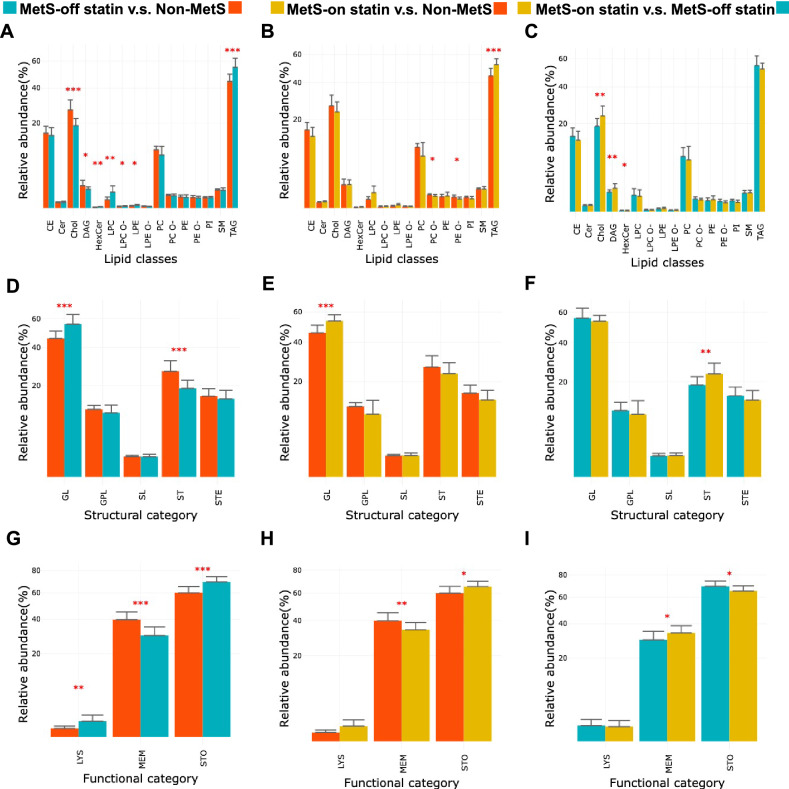
Lipid classes and structural and functional categories in VLDLs. (**A**–**C**) Relative abundance of 16 lipid classes, including cholesteryl ester (CE), ceramide (Cer), cholesterol (Chol), diacylglycerol (DAG), glucosyl/galactosyl ceramide (HexCer), phosphatidylcholine (PC), lyso-PC (LPC), phosphatidylethanolamine (PE), lyso-PE (LPE), phospholipid (PL), sphingomyelin (SM), ether derivatives of PC (PC O-), LPC (LPC O-), PE (PE O-), and LPE (LPE O-). (**D**–**F**) Relative abundance of 5 structural categories, including glycerolipid (GL), glycerophospholipid (GPL), sphingolipid (SL), sterol (ST), and sterol ester (STE). (**G**–**I**) Relative abundance of 3 functional categories, including lysolipids (LYS), membrane lipids (MEM), and storage lipids (STO). Bars for the non-MetS, MetS-off statin, and MetS-on statin groups are illustrated in red, green, and yellow, respectively.

The relative abundance of lipid components was analyzed and compared between groups with lipid classes (Fig. 3A–C), structural categories (Fig. 3D–F), and functional categories (Fig. 3G–I). The comparison between MetS-off statin and non-MetS revealed changes in MetS without the effects of statins. Sixteen lipid classes were identified, and cardiolipin was not detectable in the VLDLs of the study participants. Compared to the non-MetS group, the MetS-off statin group had a significantly greater abundance of HexCer, LPC, LPE, and TAG but less Chol and DAG in VLDL. Comparing the five lipid structural categories to non-MetS, the MetS-off statin group was more abundant with glycerolipids and less abundant with sterols (Fig. 3D). Comparing the three functional categories to non-MetS, the MetS-off statin group had more lysolipids and storage lipids and fewer membrane lipids (Fig. 3G). The comparison between the MetS-on statin and the MetS-off statin groups revealed the effects of statins (Fig. 3C,F,I). Compared to the MetS-off statin group, the MetS-on statin group had increased Chol, DAG, and reduced HexCer in lipid classes, increased sterols, increased membrane lipids, and decreased storage lipids. The comparison between the MetS-on statin and the non-MetS groups revealed residual differences in the lipid components of VLDL upon statin treatment (Fig. 3B,E,H). Compared to non-MetS, MetS-on statin still had more TAG but less PC-O and less PE-O in lipid classes, more glycerolipids in structure categories, more sterols and fewer membrane lipids in functional categories.

### Significant correlation of VLDL lipid components with LA size and function

**Figure 4:**
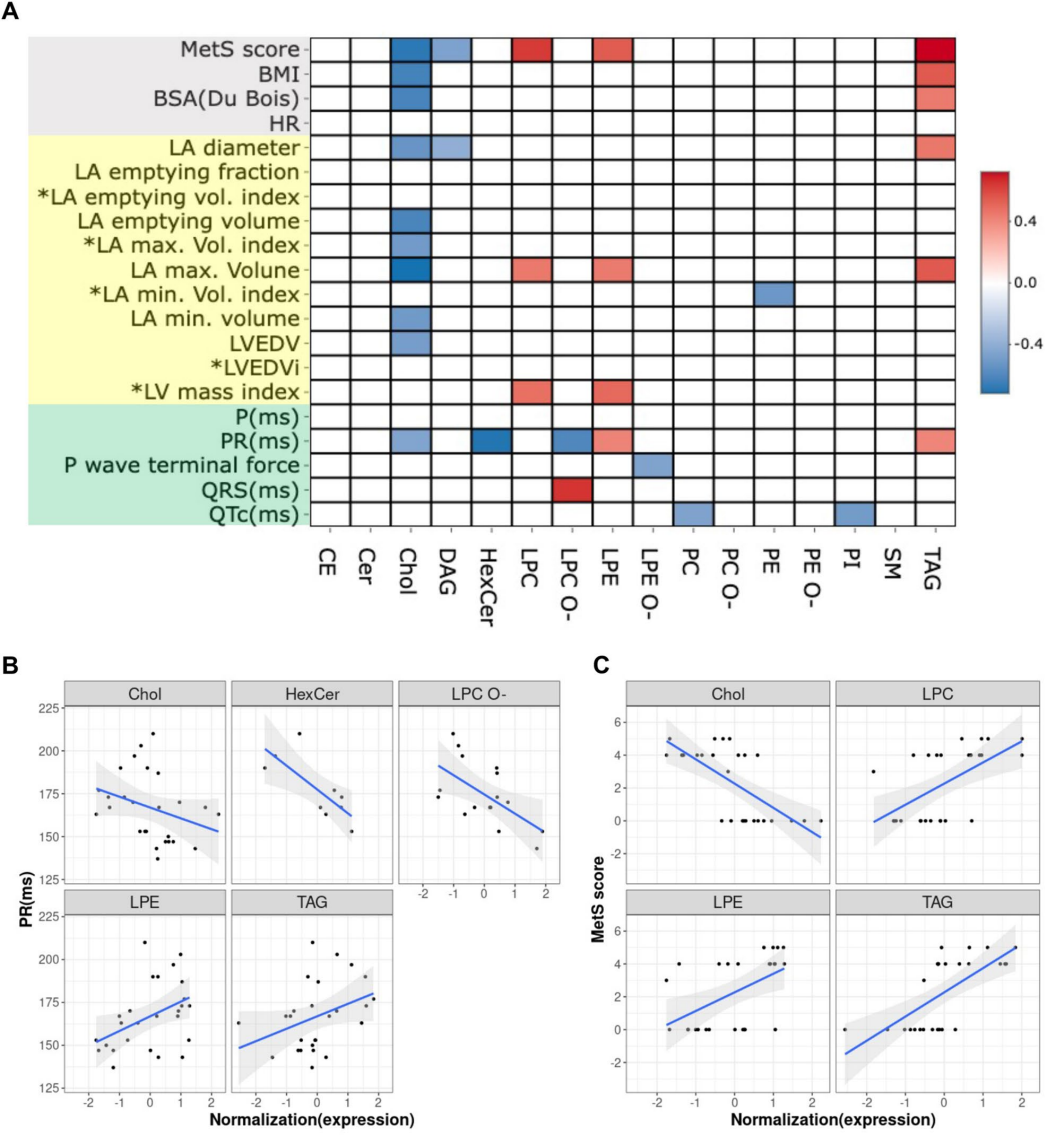
Association of VLDL lipid classes with MetS score, BMI, and cardiac remodeling markers. (**A**) Among 16 lipid classes, cholesterol (Chol), lyso-phosphatidylcholine (LPC), lyso-phosphatidylethanolamine (LPE), and triglyceride (TAG) were significantly correlated with LA size in maximum volume. (**B**) Chol, LPE, TAG, glucosyl/galactosyl ceramide (HexCer), and ether derivative of phosphatidylcholine (LPC O-) are significantly correlated with intra-atrial conduction indicated by the PR interval. (**C**) MetS score is positively correlated with LPE, LPC, and TAG but negatively correlated with Chol. Consistently, MetS score, LA maximum volume, and LV mass were positively correlated with LPC and LPE. TAG alone was positively correlated with MetS score, BMI, LA diameter, maximum volume, and PR interval.

To test the hypothesis that VLDL lipid components can affect LA remodeling, Spearman’s rank-order correlation was used to examine the correlation of each lipid class with cardiac remodeling markers, including LA diameter, maximum volume, empty fraction of LA, LV end diastole volume, and LV mass index, and parameters of electrical remodeling, such as P wave duration, PR interval, P wave terminal force, QRS width and QTc interval (Fig. 4A). Lipid classes were also analyzed for MetS scores, BMI, and HR (Fig. 4A,B). The MetS score was positively correlated with LPC, LPE, and TAG and inversely correlated with Chol (Fig. 4B). The increased diameter and maximum volume indicate LA enlargement, which is a hallmark of structural remodeling^35^. The results showed that LPC, LPE, and TAG were significantly associated with the LA maximum volume (Fig. 4A). LPE and TAG were also significantly correlated with the PR interval, indicating intra-atrial conduction velocity (Fig. 4A). The PR interval was inversely correlated with Chol, HexCer and LPC O- (Fig. 4C). LPE and LPC were also correlated with the LV mass index, which indicates ventricular hypertrophy. Consistently, MetS score, LA maximum volume, and LV mass were positively correlated with LPC and LPE. TAG alone was positively correlated with the MetS score, BMI, LA diameter, maximum volume, and PR interval. These findings together suggest that the lipid components of VLDL can affect cardiac remodeling, both the structural and electrical remodeling of LA.

### Alteration of TAG (triglyceride) total length and association of unsaturated fatty acids with cardiac remodeling

**Figure 5:**
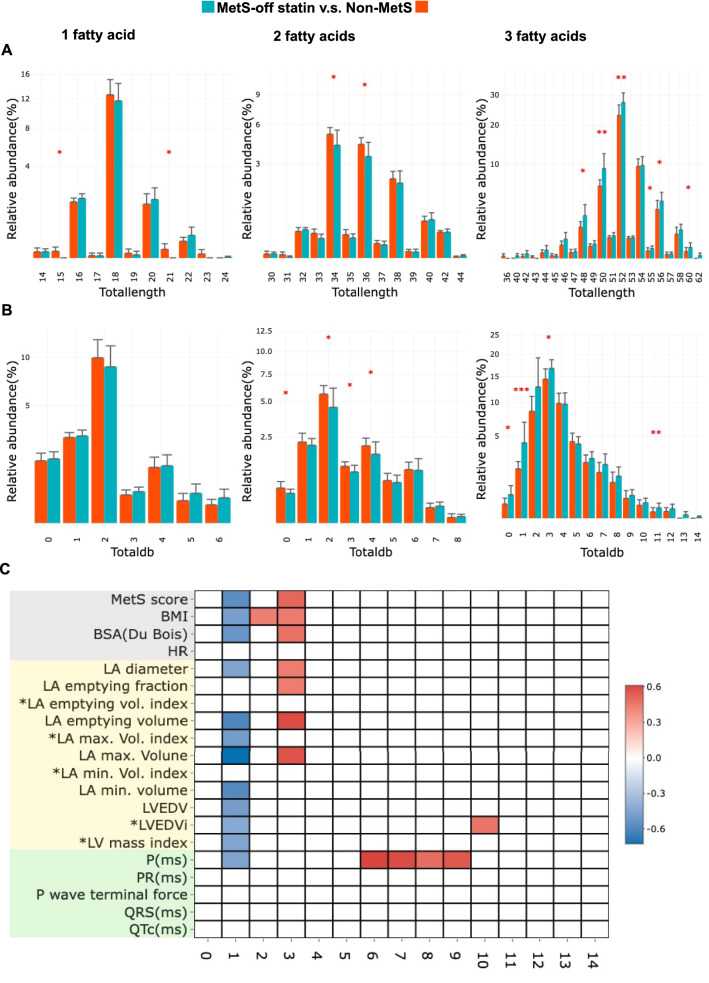
Total length and double bonds of fatty acids in VLDLs of MetS and non-MetS, and the correlation with MetS score and cardiac remodeling markers. The relative abundance of total lengths with 1, 2 and 3 fatty acids (**A**), and of double bonds in fatty acids (**B**), and the comparison between the non-MetS (red) and MetS-off statin (green) groups (**A**,**B**). (**C**) The correlation of double bond numbers with variables including MetS score, body mass index (BMI), body surface area (BSA), and markers for cardiac remodeling.

To elucidate whether any TAG changes in VLDL were correlated with MetS score and cardiac remodeling, the total length of TAG fatty acids was compared between the non-MetS and the MetS-off statin groups (Fig. 5A,B). Spearman’s correlation was used to test the correlation of the number of double bonds in TAGs with the MetS score and cardiac remodeling markers (Fig. 5C). With three fatty acids, mostly in the form of TAG, the abundance of total length with 48, 50, 52, 55, 56, and 60 carbons was greater in the MetS-off statin group than in the non-MetS group (Fig. 5A). The presence of three double bonds indicates that the unsaturated fatty acids of TAG were positively correlated with the MetS score, BMI, and LA remodeling markers, including LA diameter, maximum volume, emptying volume, and EF, that is, LA contractility. However, the presence of only a single double bond, which indicates a lower degree of unsaturated fatty acids in TAGs, was negatively correlated with MetS scores and markers for obesity and cardiac remodeling. Interestingly, the extreme richness of double bonds is positively correlated with P-wave duration, which indicates the required time for completing all myocardial action potentials in the entire and bilateral atria. This finding suggests that the richness of unsaturated fatty acids affects atrial remodeling with respect to chamber dilatation, emptying function, and regulation of action potentials.

## Discussion

The main findings from this study are as follows: (1) Compared with the VLDL of the non-MetS group, the MetS-off statin group had significantly greater lipid content with HexCer, LPC, LPC-O, LPE, and TAG and less Chol and DAG. (2) Except for TAG, increased lipid classes in the VLDL of MetS subjects were significantly attenuated by statin therapy. (3) The abundance of the lipid classes LPC, LPE, and TAG in VLDL was positively correlated with atrial dilatation, which was inversely correlated with the richness of Chol in VLDL. (4) The abundances of LPE and TAG in VLDL were significantly correlated with the PR interval. (5) Double bonds of unsaturated fatty acids were significantly correlated with atrial remodeling markers, including diameter, volume, emptying function, and P-wave duration. Together, these findings suggest that modification of lipid components in VLDL affects atrial remodeling and may be responsible for increased AF risks in MetS.

### Increased lipid components with LPC, LPE, and HexCer in VLDL and atrial remodeling

LPC is derived from the cleavage of phosphatidylcholine (PC) by phospholipase A2^36^. LPC can induce proinflammatory cytokines, oxidative stress, and apoptosis in endothelial cells and vascular smooth muscle and can also affect intracellular Ca^2+^ balance. LPC has been reported to play a role in atherosclerosis, diabetes, myocardial infarction, and Alzheimer’s disease^36, 37^. The increased LPC content in the VLDL of MetS can possibly affect atrial Ca^2+^ regulation and induce oxidative stress in atrial myocytes. This is the first study to report a correlation between LPC and atrial remodeling in MetS.

LPE originates from the exogenous lysophospholipid metabolic pathway. After uptake, lyso-PE is a major source of PE through acylation by an acyl-CoA-dependent acyltransferase Ale 1p^38^. A1e 1P activity is enriched in mitochondrial-associated membranes. The trafficking pathway for LPE to reach mitochondria remains unknown^39^. PE metabolism has been associated with Alzheimer's disease, Parkinson's disease, and nonalcoholic liver disease^36^, suggesting that the increased LPE component in VLDL may also affect mitochondrial function in atrial cardiomyocytes.

Another significantly increased lipid class in VLDL is HexCer, in which ceramide undergoes a monohexosyl substitution by specific ceramide synthases/synthases^40^. HexCer is essential for cellular physiological functions such as myelinogenesis in the nervous system, epidermal permeability, cell proliferation and apoptosis^40^. Ceramide is associated with primary cardiomyopathy and secondary cardiometabolic disorders, such as diabetic cardiomyopathy^41–43^.

While the Lands cycle has been described by William E. M. Lands^44^ as a conversion process between lysophospholipids and phospholipids, it is recognized as an important mechanism for neutral lipid consumption in cells. Lipid droplets (LDs) are the playground of the Lands cycle^45, 46^ and are colocalized in the endoplasmic reticulum (ER), where the VLDL package takes place^47^. LD served to the ER with LPC/LPE during VLDL packing. The LPC/LPE enrichment in the MetS-VLDL particle suggests a trajectory of pathological hepatocytes producing VLDL, which has been supported by other studies. In a study by Rong et al., genetic defects in lpcat3 (LD enzyme) in mice resulted in arachidonic acid, LPC, and TAG reduction in VLDL particles^48^. Hepatic lpcat3 function might occur through microsomal TAG transfer during VLDL production^49^. On the other hand, in the intestinal lumen, enhanced postprandial lysophospholipid absorption can suppress hepatic fatty acid oxidation, lead to increased VLDL synthesis and tissue lipid deposition, and improve diet-induced hyperlipidemia^50^.

### The negative correlation of cholesterol with atrial dilatation and PR interval

This study found negative correlations between cholesterol levels and atrial dilatation and the PR interval. The abundance of cholesterol in VLDL was also negatively correlated with the MetS score, BMI, LA emptying volume, and LV volume. This study finding may partially explain why cholesterol levels have a paradoxical association with incident AF in clinical observational studies^51^. Unlike the major effects of statins on the reduction of LDL cholesterol, statins increase the relative abundance of cholesterol in VLDL. Richness of TAG in VLDL particles is found destabilizing HDL from affecting CETP-induced remodeling of TAG-rich HDL, which also affects the lipolysis of VLDL^16^. The withdraw of statin led to a significant reduction of HDL (Table 1). The HDL might be counteracting the effects of LDL and TAG, dampening the overall role of cholesterol in atrial dilatation. Further studies are required to determine the role of HDL in atrial dilatation.

### Statins partially normalize lipid component changes in VLDL of MetS

In the present study, statins reduced the abnormally abundant LPC, LPC O-, LPE, and HexCer in the VLDL of MetS patients. The comparison between the two MetS indicated that statin significantly reduced long chain TAG (46:0, 46:1, 48:0, 48:1, and 50:1) in VLDL. Another study also showed that statins normalized the plasma lipidome of 12 MetS males in comparison with 12 healthy controls^52^. Consistently, statins can shift the lipid components of MetS toward being more similar to non-MetS (Fig. 2D). The reduction in LPC, which can induce inflammation and oxidative stress, may explain the mechanism underlying the anti-inflammatory effects of statins^53^. This may also partially explain the benefit of statin therapy in reducing cardiovascular events^54^. Although statins can restore the aforementioned lipid classes in MetS, TAGs remain abnormally abundant. VLDL is a major apoB-containing lipoprotein. Findings regarding the effects of statins on the VLDL lipidome are consistent with previous recognition of apoB and TAG as residual cardiovascular risk factors^55, 56^. The effects of statins on restoring VLDL lipid component changes in MetS may explain the long-term benefits of statins in reducing the risk of AF^11, 57^.

### Changes in TAGs in VLDL

Clinical observational studies have also reported elevated TAG levels in AF patients^58, 59^. This study is the first to disclose increased long-chain TAGs and the strong correlation of three double bonds of unsaturated fatty acids with atrial remodeling. This finding partially explains the increased risk of AF in patients with MetS and elevated TAG levels. The liver is the major organ that produces and secretes VLDL, suggesting that the alteration of TAG in VLDL is derived from fatty liver that commonly coexists in subjects with MetS. Nonalcoholic human fatty liver has been found to increase the activity of hepatic stearol-CoA desaturase (SCD)-1, which converts saturated fatty acids to monosaturated fatty acids as a major substrate for the synthesis of TAG and other lipids^60^. In contrast, the inverse correlation of single double bonds of fatty acids with MetS score, BMI, and almost all atrial and ventricular dilatation markers may indicate a lower degree of SCD-1 activity.

Considering the complexity of lipid metabolism, further studies are needed to elucidate the pathological role of lipid classes that are significant for atrial dilatation and conduction, including TAG. The effects of highly unsaturated fatty acids on altering atrial action potentials will be another interesting topic for studying the mechanisms^60^.

### Comparisons with other studies and what does the current work add to the existing knowledge

Techniques of lipidomics have been applied in some clinical studies for assessing the association of AF. One study found that BMI-correlated increased PC 38:3 was associated with atrial conduction, which was represented by P wave duration^61^. Another study found that AF patients had different fatty acids and phospholipids in the plasma than healthy controls^62^. Consistently, Zhou et al. found dysregulated lipid molecules in the plasma of AF patients^63^ and their study population presented a late stage of atrial remodeling. Nevertheless, long-term rhythm control of AF remains difficult, and patients at risk of AF should have effective interventions to stop the progression of atrial remodeling. Knowledge of the early stage of atrial remodeling, however, is sparse. The current work fills some knowledge gaps and impacts for the care of atrial remodeling patients.

### Study limitations

First, this is a study with a small sample size, which might lead to a high risk of type I error. The age- and sex-controlled selection of participants had reduced this limitation. Second, this study did not unify the statin regimen in the MetS-on-statin group, and the statin effects were interpreted as a class effect of drugs. Third, the blood samples were collected in the morning after an over 8 h fasting period, and the lipidome of VLDL shown in this study might not be applicable for postprandial VLDLs. Lastly, how long would a statin withdraw lead to prominent changes of VLDL lipids was not determined in this study. Since statin therapy has become an indispensable treatment for primary and secondary cardiovascular event prevention, withdrawal of statin bears an important ethical concern^64^. Our study had excluded any subjects with overt cardiovascular diseases. Data from the Treating New Targets (TNT) study suggests unincreased risk of short-term discontinuation of statin therapy in patients with stable cardiac conditions^65^, accordingly, it is assumed that 2 weeks withdraw of statin is not harmful. Nevertheless, in patients with acute coronary syndrome or ischemic stroke, stopping of statin for 3 days had been shown leading to greater myoischemia, neurological deterioration, and risk of death^66^. In an animal model, withdrawal of statins for 2 days resulted in 5- and 2.7-fold downregulation of eNOS in aorta and brain, respectively^67^. Therefore, we assumed that the absence of statin for 2 weeks is long enough to observe metabolic changes in the MetS subjects without overt cardiovascular diseases or stroke.

## Conclusions

In MetS patients with structural and electrical atrial remodeling, which indicates existing risks for AF, significantly changed components of VLDL particles carried more TAG, LPC, LPE, and PI. The significant correlation of LPC, LPE, and unsaturated fatty acids in TAGs with atrial remodeling markers further delineated the pathogenic role of VLDL lipotoxicity. The significantly lower abundance of Chol in VLDL can explain the “cholesterol paradox” in patients with paroxysmal AF^68^. Some MetS-VLDL components, such as long-chain TAG, Cer, PE, and Chol, escaped from statin benefits, suggesting a need for VLDL- and/or TAG-rich lipoprotein-targeted therapies for MetS patients with atrial remodeling to prevent AF. These results call for mechanistic studies of lipotoxicity in atrial cardiomyopathy and cardiometabolic disorders.

## Supplementary Information

Supplementary Information.

## Data Availability

All the reidentified data are available upon reasonable request (hclee@kmu.edu.tw).

## Abbreviations

AF: Atrial fibrillation
Cer: Ceramide
Chol: Cholesterol
DAG: Diacylglycerol
HexCer: Glucosyl/galactosyl ceramide
PC: Phosphatidylcholine
PE: Phosphatidylethanolamine
LPC: Lyso-PC
LPE: Lyso-PE
O-: Ether derivatives
TAG: Triacylglycerol (also named triglycerides)
VLDL: Very low-density lipoprotein
MetS: Metabolic syndrome
